# Coping in crisis: a qualitative study of stress and resilience in Ontario's long-term care workforce

**DOI:** 10.3389/frhs.2026.1789803

**Published:** 2026-05-13

**Authors:** Sheila A. Boamah, Farzana Akter, Mawukoenya Theresa Sedzro

**Affiliations:** 1 School of Nursing, Faculty of Health Sciences, McMaster University, Hamilton, ON, Canada; 2 Faculty of Health Sciences, University of Western Ontario, London, ON, Canada

**Keywords:** Canada, coping strategies, co-design, health care workforce, long-term care, resilience, stress

## Abstract

**Background:**

The global long-term care (LTC) sector is increasingly strained by entrenched structural vulnerabilities, including persistent workforce shortages, constrained resources, high resident-to-staff ratios, and rising clinical complexity, all of which compromise the delivery of safe, high-quality care. In Canada, these challenges are particularly acute, placing substantial psychological, emotional, and ethical strain on LTC workers. Despite this, empirical insight into their lived experiences, particularly within Ontario remains limited.

**Objective:**

This study explores the lived experiences of LTC staff and leaders in Ontario, Canada, examining work-related stressors and coping strategies across the acute and post-acute phases of the COVID-19 pandemic through the lens of Lazarus and Folkman's (2008) Transactional Model of Stress and Coping to inform interventions that support workforce well-being and strengthen care delivery.

**Methods:**

A qualitative co-design approach was employed using purposive and convenience sampling. Twenty-four LTC staff, including personal support workers, registered nurses, and administrators, participated in eleven focus group discussions. Guided by Experience-Based Co-Design (EBCD) principles, participants were engaged as co-researchers, contributing as collaborative partners in identifying emotionally salient “touchpoints” and jointly reflecting on workplace stressors and coping strategies. Data were thematically analyzed and subsequently mapped onto the Transactional Model of Stress and Coping, including problem-focused, emotion-focused, and meaning-focused coping strategies.

**Results:**

Five themes were identified, each reflecting an emotionally salient touchpoint and its corresponding coping response: (i) *Together in* exhaustion: solidarity through emotion-focused coping; (ii) *Silent* in grief: shared healing and meaning-focused coping*;* (iii) *Living in a prolonged emergency*: coping within institutional constraints; (iv) *Care as connection:* relational resilience through meaning-focused coping; and (v) *Reclaiming control through redefined boundaries:* problem-focused coping. Across themes, coping emerged as dynamic, relational, and co-constructed, supported by team cohesion, relational validation, and participatory leadership.

**Discussion:**

This study illuminates how LTC workers navigate emotionally salient touchpoints and systemic constraints while sustaining compassionate care through coping processes that are relational, co-constructed, and shaped by supportive interpersonal and organizational contexts. By integrating the Transactional Model with EBCD, it advances methodological and theoretical understanding of coping as a dynamic, context-embedded process rather than an individual response. These insights offer actionable directions for strengthening workforce resilience, enhancing organizational supports, and informing more responsive and relationship-centred models of care within LTC.

## 1 Introduction

Long-term care (LTC) systems worldwide have long grappled with structural fragilities, including chronic understaffing, resource limitations, and the emotional weight of caregiving, that were brought into sharp relief during recent global health crises (1). In Ontario, Canada, these systemic pressures converged to create profound strain, as frontline workers faced heightened stress, grief, and moral injury, compounded by institutional rigidity and limited organizational support (2, 3). Even amid such adversity, staff demonstrated remarkable perseverance, prompting critical questions about how resilience was maintained and which strategies enabled coping.

In LTC settings, chronic moral distress and moral injury, arising when staff must act, or witness actions, that conflict with their moral values, were particularly pronounced (4, 5). While awareness of these challenges has grown, available interventions remain limited and inconsistently implemented. Current initiatives, such as guided reflection, ethics discussions, and practical toolkits, show promise but lack the systemic integration needed for lasting impact (4). Coping in LTC, however, extends beyond individual psychological strategies; it is shaped by relational, organizational, and structural conditions that influence how stressors are interpreted and managed. Evidence from Canadian healthcare workers (HCWs) indicates that higher levels of organizational support and spiritual well-being are associated with reduced moral injury (3, 6), highlighting the critical role of supportive workplace systems in fostering resilience.

To address the multifaceted nature of stress and resilience in LTC, this study integrates the Transactional Model of Stress and Coping with Experience-Based Co-Design (EBCD)—a participatory methodology that positions participants as co-researchers, engaging them as active partners through genuine collaboration and shared decision-making to identify emotionally salient “touchpoints” and co-develop improvements (7, 8). Within Ontario's LTC sector, EBCD has already demonstrated considerable value: co-design initiatives have yielded practical engagement toolkits and have been explicitly deployed to illuminate the roots of burnout while co-creating supportive interventions with LTC staff (9, 10). Although the psychological toll of the COVID-19 pandemic on HCWs is well established (11, 12), few studies have examined LTC staff coping through a theoretically informed framework that bridges cognitive-emotional processes with organizational and system-level dynamics.

In response, this study undertakes a thematic analysis of focus groups with Ontario LTC workers to: (1) identify the primary stressors encountered during and after the pandemic; (2) categorize coping strategies into problem-focused, emotion-focused, and meaning-focused modalities; (3) explore how systemic and relational factors, including leadership, team climate, and organizational supports, shape resilience; and (4) generate evidence-based insights to inform interventions and policy reforms aimed at fortifying workforce resilience in the post-pandemic era.

By foregrounding the lived experiences of LTC workers and situating coping strategies within their structural, emotional, and participatory contexts, this research contributes a theoretically nuanced yet practice-oriented account of resilience in LTC—one that seeks to bridge individual adaptation with collective transformation.

## 2 Methods

### 2.1 Study design

This paper presents findings from a qualitative analysis embedded within a broader multi-phase, mixed-methods study exploring mental health resilience and workforce experiences among LTC workers in Ontario, Canada. A qualitative approach was adopted to enable a deep, context-rich understanding of participants' coping narratives, illuminating the emotional and relational dimensions of caregiving under crisis conditions that are often obscured in quantitative designs.

The overarching study design and methodological framework are described in detail in Boamah et al. (9), which examined workforce challenges and the co-development of solutions in LTC settings. Drawing specifically from Phase 1 of the project, this paper offers a distinct, in-depth exploration of the coping mechanisms and strategies LTC workers employ in response to sustained moral stress and systemic pressures.

### 2.2 Co-design framework and relevance

The co-design component of this study was guided by the EBCD approach (13), emphasizing participatory, person-centered engagement and reciprocal learning between researchers and LTC staff. As detailed in Boamah et al. (14), EBCD was selected for its ability to capture lived experiences while fostering the collaborative development of actionable interventions. This participatory approach is particularly well-suited to the LTC context, characterized by chronic understaffing, moral distress, and burnout, as it offers a framework for examining systemic stressors and individual coping strategies in an integrated and context-sensitive way.

### 2.3 Theoretical framework

The theoretical foundation of this study was anchored in Lazarus and Folkman's (15) (2008) Transactional Model of Stress and Coping, a well-established framework in health and organizational psychology. The model conceptualizes stress as a dynamic process arising from interactions between individuals and their environment, mediated through cognitive appraisal. Stress occurs when environmental demands are perceived as exceeding one's coping resources, involving two interrelated evaluative processes: ***primary appraisal*** (assessing the significance and potential harm or challenge of a stressor) and ***secondary appraisal*** (evaluating available coping resources and possible responses).

Within the Transactional Model, coping is viewed not as a fixed trait but as an adaptive and evolving process. Accordingly, three primary coping strategies were integrated into the analytical framework of this study:

#### 2.3.1 Problem-focused coping

Active efforts to modify or manage the source of stress, such as direct problem-solving, seeking practical support, or restructuring tasks.

#### 2.3.2 Emotion-focused coping

Strategies aimed at regulating emotional responses to stressors, including emotional expression, seeking emotional support, distancing, or avoidance.

#### 2.3.3 Meaning-focused coping

Introduced by Folkman (16), this form of coping involves drawing upon existential beliefs, personal values, and identity to derive purpose, sustain motivation, and maintain psychological balance under persistent or uncontrollable stress.

The selection of this framework was informed by its extensive empirical validation, conceptual flexibility, and strong applicability to healthcare contexts, where professionals often contend with chronic stress, ethical tensions, and complex emotional demands (15, 17). Within LTC settings, workers face sustained exposure to moral injury, trauma, loss, and limited institutional control, necessitating a broad and adaptive coping repertoire that extends beyond immediate problem-solving. The Transactional Model's dynamic appraisal processes thus provided a robust lens through which to examine how LTC workers continually evaluated their circumstances and refined coping strategies over time, offering both theoretical depth and analytical clarity to this study.

### 2.4 Conceptual–methodological alignment

To deepen the inquiry and strengthen its contextual relevance, the theoretical framework was integrated with the EBCD—a participatory approach that foregrounds experiential knowledge, emotional insight, and collaborative sense-making (18). EBCD emphasizes participant engagement and the identification of emotionally salient “touchpoints” that shape daily care experiences. This approach aligns closely with the Transactional Model's focus on subjective appraisal and person–environment interaction, offering a methodological bridge through which coping can be examined as both an individual strategy and a socially mediated process.

In practice, EBCD enabled a dialogic exchange between researchers and LTC workers, allowing participants to reflect on coping practices, evaluate institutional contexts, and co-create strategies for psychological resilience. This participatory process amplified participants' voices, illuminating how structural factors, interpersonal dynamics, and professional identity converge to shape responses to stress. The integration of EBCD with the Transactional Model thus positioned coping not merely as an individual cognitive process but as an emergent outcome of shared meaning-making and systemic negotiation.

### 2.5 Participant recruitment and sampling

Participants were recruited using purposive and convenience sampling to capture diversity across occupational roles, care settings, and sociodemographic backgrounds. Eligibility required participants to have worked in LTC homes for at least six months during the COVID-19 pandemic. Recruitment strategies included digital flyers distributed via professional associations, social media outreach (e.g., X/Twitter™), and webinar-based information sessions. Participants included personal support workers (PSWs), registered nurses, leaders/administrators, and social workers. The heterogeneity of this sample enhanced analytical depth and supported theoretical saturation by encompassing a broad spectrum of coping behaviours and contextual influences. Additional methodological details are provided in the study protocol published elsewhere (9).

### 2.6 Data collection

Data were collected between July 2023 and October 2024 through eleven focus group discussions, conducted by the lead author either virtually or in person, depending on participant availability and location. Five exploratory sessions captured LTC workers' lived experiences during and after the pandemic, while six subsequent sessions focused on co-developing practical coping strategies within the workplace. An interview guide structured the discussions and included demographic questions (e.g., age, gender identity, role/job title, years of practice experience), participants' experiences as healthcare workers, current challenges (e.g., changes in work and life since the pandemic), and coping strategies (e.g., methods enabling them to navigate challenges in LTC settings), which were collected prior to the focus group sessions to inform and guide the subsequent discussions. To promote psychologically safe and open dialogue, breakout groups were organized by staff role (e.g., PSWs, administrators, nurses) to minimize professional hierarchies. Each two-hour session was facilitated by the principal investigator, supported by a trained co-facilitator and notetaker. Discussion guides, developed collaboratively with LTC sector advisors, addressed mental health challenges, organizational supports, psychological safety, and resilience at both individual and collective levels. All sessions were audio- and video-recorded with participant consent and transcribed verbatim by two research team members. Identifying information was removed, and data analysis was conducted on de-identified transcripts using Delve qualitative data analysis software.

### 2.7 Data analysis

Data were analyzed thematically following Braun and Clarke's (19) reflexive six-phase framework: 1) data familiarization, 2) open coding of transcripts, 3) generation of initial themes, 4) theme development through reflexive discussions, 5) review and refinement of themes, and 6) final articulation. Transcripts were analyzed using a two-tiered process: an inductive phase to identify emergent patterns in participants' narratives, followed by a deductive phase aligning these themes with the Transactional Model's dimensions of problem-focused, emotion-focused, and meaning-focused coping. Analytical rigor was ensured through iterative coding, team-based discussions, and reflexive memo-writing to capture interpretive decisions and researcher perspectives. Member-checking with a subset of participants further validated thematic interpretations and enhanced credibility. By integrating inductive and deductive strategies, the analysis was both grounded in participants' experiences and theoretically informed, illuminating how LTC workers navigated stress, sustained resilience, and constructed meaning within morally and institutionally complex environments.

### 2.8 Reflexivity

Reflexive practices were embedded throughout the study to address the influence of researchers' perspectives on data collection and analysis (20). These included analytic memoing, monthly reflective team discussions, collaborative interpretation, and peer debriefing, including multiple transcript reviews, to ensure credibility and dependability. Investigator triangulation drew on the interdisciplinary expertise of the team in LTC and health workforce research. EBCD further supported reflexivity by involving participants in shaping interpretations, ensuring findings remained grounded in their lived experiences.

### 2.9 Ethical considerations

Ethics approval for the study was granted by the Hamilton Integrated Research Ethics Board (HIREB #14837). The study adhered to the Tri-Council Policy Statement (TCPS2, 2018) and the Declaration of Helsinki. All participants provided written informed consent and were assured of confidentiality and the right to withdraw without consequence.

## 3 Results

Based on thematic analysis of 11 focus group discussions with 24 LTC workers across Ontario, five key themes emerged, highlighting how participants appraised stressors and employed coping strategies during and after the COVID-19 pandemic. Anchored in Lazarus and Folkman's Transactional Model of Stress and Coping (16) and its later refinement (21), each theme was interpreted through the lens of primary and secondary appraisal and categorized according to the coping strategy employed: problem-focused, emotion-focused, or meaning-focused (see Table 1).

**Table 1:** Thematic summary of stress appraisal and coping strategies among long-term care workers.

Themes	Situation/Challenges Faced	Cognitive Appraisal	Coping Strategy
“Together in Exhaustion”: Solidarity Through Emotion-Focused Coping	Overwhelming workloads, burnout, emotional labor, and insufficient systemic support, Limited formal psychosocial supports; pressure to “hold the line”	**Primary Appraisal:** Participants appraised the working environment as emotionally threatening and overwhelming. Burnout was seen as a collective rather than individual phenomenon, characterized by unrelenting stress, physical exhaustion, and emotional strain.	**Emotion-focused coping:** emotional sharing, humor, mutual empathy, and informal check-ins to process their stress and maintain emotional stability.
		**Secondary Appraisal:** While institutional resources were perceived as inadequate, participants felt they had relational resources (e.g., peer support) to mitigate distress.	
“Silent Grief, Shared Healing”: Emotion- and Meaning-Focused Coping	Repeated exposure to death and lack of time or structure for formal grieving.	**Primary Appraisal:** The frequent deaths of residents and colleagues were appraised as irreversible loss and harmful events, evoking deep emotional trauma.	**A blend of emotion-focused and meaning-focused coping:** Emotionally, participants created informal rituals (e.g., memorial gardens) and supportive spaces to express grief. Simultaneously, they engaged in meaning-making by reframing their caregiving role as a source of purpose and identity.
		**Secondary Appraisal:** Participants perceived limited organizational support for grief processing, with inadequate access to counseling or structured mourning rituals.	
“Living in a Prolonged emergency”: Coping within Institutional Constraints	Chronic enforcement of emergency mandates and constant institutional monitoring created emotional fatigue and alienation.	**Primary Appraisal:** Participants perceived institutional rigidity—prolonged emergency protocols, communication failures, and administrative detachment—as persistent stressors.	**A combination of problem-focused and emotion-focused coping:** Emotion-focused strategies included venting, peer solidarity, and psychological distancing.
		**Secondary Appraisal:** Control over systemic change was seen as limited; however, participants identified small opportunities for local-level adaptation and advocacy.	Problem-focused strategies emerged through informal policy discussions, advocacy for clearer communication, and streamlining practices at the unit level.
“Care as Connection”: Relational Resilience through Meaning-Focused Coping	Emotional labor in absence of formal recognition or external validation.	**Primary Appraisal:** Providing care to isolated residents was emotionally intense but also experienced as deeply meaningful. The stress stemmed from emotional depletion and lack of systemic recognition.	**Meaning-focused coping**: Participants derived existential fulfillment and personal identity affirmation from caregiving, reframing their emotional labor as a form of moral commitment
		**Secondary Appraisal:** Participants drew strength from resident relationships and family appreciation, enabling them to sustain caregiving roles.	
“Reclaiming Control through Redefining Boundaries”: Problem-Focused Coping	Blurred personal-work life boundaries and emotional burnout among leaders and frontline workers.	**Primary Appraisal:** The collapse of work-life boundaries was appraised as a long-term threat to mental health and role sustainability.	**Problem-focused coping:** built-in vacation time, wellness spaces, and performance-based well-being indicators. Administrators modeled emotional transparency and vulnerability to create psychological safety and reinforce trust.
		**Secondary Appraisal:** Leaders and staff believed they had the capacity to enact localized structural changes and promote wellness.	

### 3.1 “Together in exhaustion”: solidarity through emotion-focused coping

A salient theme across focus group discussions was the shared experience of chronic emotional exhaustion, driven by relentless workloads, the emotional demands of caregiving, and a persistent lack of systemic support. Participants consistently appraised their work environments as emotionally threatening, characterizing conditions as unsustainable and psychologically taxing. Through primary appraisal, these circumstances were perceived as harmful and overwhelming; secondary appraisal revealed limited perceived control over entrenched structural stressors. This imbalance shifted coping efforts away from problem-focused strategies toward relational and emotion-focused responses.

Rather than withdrawing or disengaging, participants described intentionally cultivating emotionally safe, collegial spaces in which stress could be collectively acknowledged and processed. These shared environments fostered mutual empathy, validation, and understanding, enabling participants to regulate emotional distress while maintaining professional functioning. Such practices functioned as central emotion-focused coping strategies within the Transactional Model of Stress and Coping.

Informal, peer-based interactions, including emotional sharing, humour, and communal normalization of distress, were repeatedly mobilized to restore emotional equilibrium. Participants emphasized that coping was inherently relational, with individual well-being deeply intertwined with team cohesion and the capacity to deliver compassionate care. In this way, solidarity itself emerged as a critical protective resource, buffering emotional exhaustion in the absence of adequate institutional support. As one manager articulated the dual responsibility inherent in their role: “*We keep residents safe and alive and, have some semblance of quality of life, while we make sure that we’re looking after our employees at the same time, so it was a really tricky balance.”* (Manager 01, female). Several participants underscored the necessity of emotional and physical replenishment as a prerequisite for effective caregiving, reflecting appraisal processes central to the model. One participant noted: “*We are really of no use to anybody when we are not rested, when we are not in a good space ourselves.”* (Behavioral Therapist 01, male). Others described how emotional strain propagated across organizational roles, illustrating the cyclical and cumulative nature of stress when coping resources were depleted. As one assistant director observed: “*The staff, I feel, are burnt out and looking to management for some support, but management themselves are burnt out. And it's a cycle.”* (Assistant Director of Nursing 01, female).

These narratives illustrate that coping strategies were relationally constructed, not merely through internal emotional regulation, but through acts of co-presence, shared vulnerability, and mutual affirmation. In this context, emotion-focused coping was not a passive process from stress, but an active process of collective adaptation, allowing workers to endure the emotional weight of caregiving during an unprecedented crisis.

### 3.2 “Silent grief, shared healing”: emotion- and meaning-focused coping

Participants described repeated and often abrupt exposure to resident and colleague deaths, frequently occurring without formal institutional mechanisms for grief processing. These losses were appraised as traumatic stressors, leaving enduring emotional residue and moral distress. Emotion-focused coping manifested through silent grieving and shared acknowledgment of loss, while meaning-focused coping emerged through acts of remembrance, ritual, and symbolic commemoration. One participant reflected on the unresolved nature of this grief: *“We didn’t even have time to say goodbye. One day, they were there: the next, gone. It still hurts me, years later.”* (RPN 02, female). Others described collective efforts to preserve dignity and memory amid ongoing loss. As one assistant director explained: “*We have developed a beautiful [memorial] throughout the COVID pandemic of all of the residents that we have lost and our staff that we have lost… we have created a kind of a garden of honor for them”* (Assistant Director of Nursing 01, female). Several participants articulated carrying a persistent emotional burden that accumulated over time. One PSW described this as a form of lingering trauma: “*You’re always reliving it all day long, and then you go home, and you think about it again”* (PSW 01, female).

These accounts illustrate that grief extended beyond individual deaths to encompass the loss of normalcy, routine, and human connection. For some, meaning was restored through ritualized remembrance; for others, coping took the form of emotional numbing or quiet withdrawal. Collectively, these responses reveal resilience as a relational and non-linear process—one rooted in shared acknowledgment rather than resolution.

### 3.3 “Living in a prolonged emergency”: coping within institutional constraints

The third theme captures how participants appraised ongoing COVID-era restrictions, including mandatory masking, heightened surveillance, and visitor limitations, as a chronic institutional stressor. These measures were experienced as organizational rigidity that persisted beyond the acute phase of the pandemic, contributing to emotional strain and professional isolation. One participant described the cumulative impact of unrelenting procedural demands: “*We kept getting new memos, new rules, new acronyms… but no one asked how we were doing. Just keep going, keep surviving”* (Behavioral Therapist 01, male). Administrators/leaders similarly highlighted the psychological toll of sustained surveillance protocols. One noted: “*We're still required under heightened surveillance… there's this element of protection that still seems to be looming over long-term care when everything else around us is changing”* (Administrator 01). This disconnect between external progress and internal stagnation intensified stress among frontline staff. As one assistant director explained: “*We're still wearing masks; you're still considering every move you make… The staff feel more monitored. It grates on them, and it creates a difficult work environment”* (Assistant Director of Nursing 02, female).

Participants further emphasized that frequent policy changes, often introduced without sufficient training or orientation, left both new and experienced staff overwhelmed: *“There's so much they have to learn… policies, procedures—it's all of those things. It's very overwhelming in long-term care”* (Manager 02, female). Despite limited control over institutional mandates, workers employed a combination of problem- and emotion-focused coping strategies. These included informal advocacy for clearer communication, peer consultations, venting frustrations, and collective sense-making. While conditions remained largely unchanged, these strategies enabled endurance within a perceived state of perpetual crisis. Resilience, in this context, was marked not by optimism, but by perseverance in the absence of alternatives.

### 3.4 “Care as connection”: relational resilience through meaning-focused coping

Caregiving relationships were appraised as emotionally demanding yet profoundly meaningful. Meaning-focused coping was especially salient in this theme, as participants reframed their professional identities through deep relational bonds with residents. Emotionally intense encounters were commonly described as heavy but purposeful, reinforcing values of compassion, presence, and relational care. One participant shared: “*I love your grandma like your mom, probably as much as you do… and I cry when they go”* (PSW 01, female). Another reflected on assuming familial roles during periods of isolation: “*We were their family through the pandemic… we made sure that she got a big hug every day. We did our best”* (PSW 01, female). Even small gestures of recognition helped sustain emotional investment. As one nurse noted: “*Only families… will give you a Christmas card saying thank you”* (RPN 01, female).

These moments of acknowledgment reinforced meaning amid emotional exhaustion and institutional invisibility, enabling participants to sustain engagement despite cumulative stress.

### 3.5 “Reclaiming control through redefined boundaries”: problem-focused coping

Leaders and frontline staff alike described how prolonged crisis conditions eroded personal–professional boundaries. Being “always on” became unsustainable, prompting leaders and frontline staff to appraise boundary loss as a modifiable stressor. In response, problem-focused coping strategies emerged that prioritized rest, disconnection, and intentional boundary-setting. One administrator explained: “*For the first time in my career, I put my phone on sleep… I need to decompress. As a leader, we have to demonstrate those boundaries”* (Administrator 01, female). Others described deliberate efforts to disengage from work outside scheduled hours: “*I didn’t look at my work email all weekend… that was a big step for me”* (Assistant Director of Nursing 01, female). Transparency and emotional honesty from leadership further contributed to trust and psychological safety. As one participant noted: “*I always valued transparency… The trust that the staff had was a lot higher”* (Assistant Director of Nursing 01, female). Leaders also advocated for tangible wellness supports. One described efforts to create restorative staff spaces: *“We’re putting together a back patio… plants and flowers to make that space inviting for them to shut it off”* (Assistant Director of Nursing 02, female). Together, these strategies reflect a shift from individual endurance toward structural and relational interventions that support sustainable coping and organizational resilience.

## 4 Discussion

This study explored the lived experiences and coping responses of participating LTC staff and leaders in Ontario, Canada during the acute and post-acute phases of the COVID-19 pandemic. Guided by Lazarus and Folkman's Transactional Model of Stress and Coping and Folkman's extension to meaning-focused coping, the findings extend this framework by illustrating how workers appraised and adapted to prolonged moral distress, cumulative grief, and organizational strain. Coping emerged not as a fixed individual capacity, but as a dynamic, context-dependent process shaped by the interaction between personal resources and institutional conditions. While problem- and emotion-focused strategies provided situational protection against immediate stressors, meaning-focused coping was central to sustaining resilience, preserving professional identity, and maintaining moral coherence under chronic strain. By situating coping within the relational and organizational realities of LTC work, this study advances understanding of stress appraisal in high-intensity care environments and underscores the limits of individual resilience in the absence of structural support.

The first theme, “*Together in Exhaustion*,” illustrates how coping was experienced and enacted collectively rather than individually. Consistent with findings from systematic reviews and meta-analyses, participants described persistent burnout, moral fatigue, and role devaluation, reflecting shared appraisals of threat and constraint shaped not by individual vulnerability but by entrenched structural and organizational conditions, including chronic understaffing, limited resources, and sustained exposure to resident suffering (22–24). Viewed through an EBCD lens, these stressors were collectively interpreted and negotiated within teams, shaping the moral and emotional terrain of care work and reinforcing enduring patterns of distress beyond the acute phase of the pandemic. Despite these pressures, workers drew on peer solidarity, humour, and mutual validation as forms of collective resilience, sustaining team functioning during prolonged crises (Grailey et al., 2021) (25, 26),.

Not all social strategies, however, were uniformly protective. Quantitative evidence during COVID-19 indicates that seeking social support can, paradoxically, be associated with higher perceived stress among HCWs (27, 28)—a pattern likely reflecting help-seeking behaviours that occurs under already heightened strain. In contrast, avoidant coping consistently predicts poorer mental health outcomes, including higher depression scores and reduced professional quality of life (23, 29). These findings align with the Transactional Model of Stress and Coping, highlighting that the adaptiveness of coping depends on congruence between strategy and perceived controllability: under low-control, high-demand conditions/stressors, emotion-focused or avoidant responses may predominate, but are less likely to be adaptive.

The second theme, “*Silent Grief, Shared Healing*,” captures participants’ narratives of recurrent loss, restricted family presence, and ethically constrained caregiving, highlighting the enduring nature of grief and moral distress in LTC. Consistent with prior scholarship, moral distress arises when healthcare providers are unable to act in accordance with ethical or professional commitments due to structural or institutional barriers (2, 30). Using the Transactional Model of Stress and Coping, these experiences reflect ongoing appraisals of ethical threat and constraint, while an EBCD-informed analysis reveals how staff collectively engaged in relational and symbolic coping. Peer-led dialogue, storytelling, and shared remembrance rituals functioned as informal yet deeply restorative mechanisms, supporting communal meaning-making and fostering collective moral resilience (25). Evidence from scoping reviews and qualitative studies indicates that formal grief and bereavement supports for staff remain scarce (5, 31), and in their absence, workers often rely on peer-initiated practices—closely mirroring the co-created strategies observed in this study (31, 32). Canadian toolkits and practice guidelines similarly advocate accessible, unit-based memorial practices, such as reflection rooms, dignity walks, and memory trees, to acknowledge loss and cultivate communal healing (33), reinforcing the relevance and applicability of the organically developed relational coping strategies identified in this study.

Building on the collective coping and relational resilience highlighted above, the third theme, “*Living in a Prolonged Emergency,”* captures participants' experiences of enduring crisis, marked by delayed policy recalibration and chronic staffing shortages that persisted long after the acute pandemic waves had subsided. This sentiment aligns with international research documenting how facility- and community-level factors, including workforce depletion, policy disconnects, and fragmented decision-making, undermined outbreak responses and constrained local problem-solving (34, 35). In Ontario and across Canada, pre-existing systemic vulnerabilities, including understaffing, weak integration with primary and specialty care, and fragile infection prevention infrastructures, amplified pandemic harms and complicated recovery (24). Viewed through a transactional lens, participants collectively interpreted these low-control, externally driven stressors, engaging in relational coping strategies—peer support, shared problem-solving, and team-based meaning-making—to buffer threat appraisals and sustain psychological equilibrium when problem-focused solutions were limited.

A key contribution of this study lies in the fourth theme, “*Care as Connection*,” which captures participants' redefinition of caregiving relationships as enduring sources of meaning, pride, and professional identity. Viewed through Folkman's (21) framework of meaning-focused coping, these relational experiences generated positive emotions that helped sustain engagement under chronic, uncontrollable stressors. Empirical research similarly links benefit-finding and positive reappraisal to enhanced well-being during prolonged adversity (36, 37). Within LTC, qualitative studies highlight the coexistence of joy and grief in caregiving, illustrating how relational bonds buffer strain and promote workforce retention—patterns that align with participants' accounts. Using an EBCD lens, these coping processes were not solely individual but co-constructed through team interactions, storytelling, and shared reflection, translating relational experiences into collective sources of resilience.

Finally, the fifth theme, “*Reclaiming Control through Redefined Boundaries*,” extends this framework, highlighting participants' proactive boundary-setting and restorative leadership strategies. These included modeling rest, normalizing help-seeking, and creating wellness spaces—team-informed practices that foster psychological safety and reduce stress (14, 38), (Grailey et al., 2021). Framed through the Transactional Model of Stress and Coping, these strategies reflect adaptive problem- and meaning-focused responses to chronic, low-control stressors, while an EBCD perspective demonstrates how frontline insights and relational coping can be translated into sustainable, theory-informed organizational change, reinforcing both individual and collective resilience under systemic pressures (7). Global policy guidance similarly emphasizes organizational responsibility for staff well-being, cautioning against overreliance on individual “grit” (39, 40).

### 4.1 Implications of the study

This study highlights the need to reconceptualize coping in high-stress healthcare environments. Rather than framing coping as an individual responsibility, the findings reveal it as a collective, relational, and structurally mediated process, shaped by organizational conditions, leadership practices, and the dynamics of workplace relationships. By conceptualizing moral resilience as relational and system-level, the study challenges workforce policies that focus narrowly on individual self-care while neglecting the institutional factors that produce sustained moral strain. These insights have direct implications for organizational leadership and health system governance. Supporting workforce well-being requires moving beyond traditional resilience training toward embedded structural supports that promote collective coping and moral repair. Practical examples include protected time for staff reflection, facilitated peer dialogue, and organizational rituals that acknowledge loss and ethical burden (41, 42). Integrating these practices into routine operations, rather than offering them as optional supports, signals institutional accountability for the moral and emotional dimensions of care work and fosters a culture of sustained workforce resilience.

Specific strategies include peer-led debriefing forums, such as *Schwartz Rounds,* which provide structured, multidisciplinary spaces for staff to process emotional and ethical challenges, reducing psychological and moral distress (41, 42). Unit-based memorial practices, such as dignity walks, memory trees, and reflection rooms, offer tangible rituals that honor loss, strengthen peer bonds, and promote communal healing (33). Additional measures may involve assigning peer-support champions on each unit and integrating moral resilience training into leadership development and onboarding programs. At a broader policy level, recognizing collective coping as integral to workforce sustainability and retention can inform standards for staffing, workload, psychological safety, and ethical practice, particularly in LTC settings. Embedding expectations for relational and ethical support within accreditation and regulatory frameworks shifts responsibility for workforce well-being from individual staff to organizations and systems.

A growing body of international evidence highlights that strengthening LTC systems requires the deliberate integration of relational and emotional dimensions of care into system design. Key mechanisms include fostering workforce autonomy, ensuring continuity of care, and embedding comprehensive psychosocial supports. Exemplars of this approach include the relationship-centered *Buurtzorg Model* in the Netherlands—grounded in a “humanity over bureaucracy” ethos with self-managing teams and person-centered nursing—as well as workforce well-being initiatives within the UK National Health Service and pandemic-informed reforms in Canada and Australia. Together, these cases underscore that acknowledging and supporting emotional labour is central to sustaining the workforce, delivering high-quality care, and enhancing system resilience (43–45).

Theoretically, the study extends Folkman's expanded transactional coping framework by demonstrating how problem, emotion-, and meaning-focused coping strategies co-exist and are shaped by organizational constraints, resource scarcity, and leadership practices in LTC. Methodologically, engaging LTC staff as co-researchers—active partners in the research process—illuminates the contextual, symbolic, and relational mechanisms of adaptation, while participatory approaches serve both as inquiry and intervention, supporting institutional learning, ethical accountability, and systemic resilience. From a research and implementation standpoint, future studies should evaluate system-level interventions using participatory, longitudinal, and mixed-methods designs to examine how leadership decisions, governance structures, and policy environments shape coping trajectories over time. Approaches like EBCD can ensure that interventions are grounded in frontline realities, fostering collective resilience, moral repair, and actionable knowledge for organizational and policy change.

### 4.2 Strengths and limitations

A key strength of this study is the integration of Lazarus and Folkman's Transactional Model of Stress and Coping with EBCD, offering a theoretically grounded and methodologically innovative approach to understanding coping within LTC. By positioning participants as co-researchers, contributing as collaborative partners, the study explicitly challenges hierarchical models of knowledge production and affirms lived experience as a legitimate source of epistemic authority. This participatory orientation enables the co-construction of rich, context-specific insights into the emotional, relational, and moral dimensions of caregiving during and immediately following the COVID-19 pandemic. The inclusion of diverse LTC roles, including PSWs, nurses, administrators/leaders, and social workers, enhances analytic depth and supports the transferability of findings across similar institutional contexts. Thick description, supported by participant narratives and quotations, illuminates how systemic constraints shape coping practices, while the findings generate actionable implications for organizational and policy-level intervention.

The findings should be interpreted in light of a few limitations. The use of purposive and convenience sampling, combined with a relatively small qualitative sample, may limit the transferability of findings to other contexts, and the cross-sectional design precludes examination of how coping processes change over time. Data collection occurred during 2023–2024, a period marked by sustained workforce strain, which constrained participation. Although focus groups were supplemented with asynchronous engagement opportunities, time pressures and technological barriers may have influenced the depth of participation. Future research would benefit from longitudinal and comparative designs, broader geographic sampling, and individual interviews to further examine the dynamic and structural nature of coping among LTC workers.

## 5 Conclusion

This study demonstrates that coping in LTC during prolonged crises is dynamic, relational, and systemically shaped, involving problem-, emotion-, and meaning-focused strategies enacted through peer support, leadership responsiveness, and empathetic connections with residents. The findings highlight the power of participatory, narrative-driven approaches to reveal emotional touchpoints and co-produce actionable knowledge. In this context, resilience emerges not as mere endurance, but as a process of reclaiming agency, reconstructing meaning, and enabling collective moral repair. These insights point to the urgent need for organizational and policy responses that embed emotional infrastructure, ethical leadership, structured opportunities for peer reflection, and collaborative design into the fabric of long-term care. Positioning workforce well-being as a system-level responsibility is essential, not only to sustain those who provide care, but to ensure that resilience is collective, enduring, and meaningfully enacted in practice.

## Data Availability

The raw data supporting the conclusions of this article will be made available by the lead author, upon reasonable request.
